# Obsessive-compulsive personality disorder symptoms as a risk factor for postpartum depressive symptoms

**DOI:** 10.1007/s00737-018-0908-0

**Published:** 2018-08-31

**Authors:** Kiki E. M. van Broekhoven, Annemiek Karreman, Esther E. Hartman, Paul Lodder, Joyce J. Endendijk, Veerle Bergink, Victor J. M. Pop

**Affiliations:** 1 Department of Medical and Clinical Psychology, Tilburg University, Warandelaan 2, 5037 AB, PO Box 90153, 5000 LE Tilburg, The Netherlands; 2 Department of Medical and Clinical Psychology and Department of Methodology and Statistics, Tilburg University, Warandelaan 2, 5037 AB Tilburg, The Netherlands; 3 Department of Social and Behavioural Sciences, Universiteit Utrecht, Heidelberglaan 1, 3584 CS Utrecht, The Netherlands; 4 Department of Psychiatry, Erasmus MC, Wytemaweg 80, 3015 CN Rotterdam, The Netherlands

**Keywords:** Obsessive-compulsive personality disorder symptoms, Personality, Postpartum depressive symptoms, Trajectories

## Abstract

For women with obsessive-compulsive personality disorder (OCPD) trait symptoms, coping with childbearing and parenting could be associated with postpartum depressive symptoms. Therefore, the possible relationship between OCPD trait symptoms and trajectories of postpartum depressive symptoms was examined. A cohort of 1427 women was followed from late pregnancy until 12 months’ postpartum. Trajectories of postpartum depressive symptoms were determined using growth mixture modeling with five repeated assessments. Next, the relationship between OCPD trait symptoms and these trajectories was examined through multinomial regression. Three postpartum depressive symptom trajectories were identified: (1) low symptoms (92%), (2) increasing-decreasing symptoms (inverted u-shape) (5%), and (3) increasing symptoms (3%). OCPD trait symptoms were associated with a higher likelihood of the trajectories increasing-decreasing symptoms (OR 1.26; 95% CI 1.14–1.39) and increasing symptoms (OR 1.16; 95% CI 1.02–1.32), compared to reference trajectory (low symptoms), adjusted for age, educational level, unplanned pregnancy, previous depressive episode (s), and parity.

**Electronic supplementary material:**

The online version of this article (10.1007/s00737-018-0908-0) contains supplementary material, which is available to authorized users.

## Introduction

Obsessive-compulsive personality disorder (OCPD) is one of the most common personality disorders in the general population (3–8%) (Diedrich and Voderholzer 2015) but has almost never been studied during the perinatal period (Akman et al. 2007). OCPD is defined as a relatively stable, pervasive pattern of preoccupation with orderliness, perfectionism, and mental and interpersonal control. It is indicated by symptoms such as perfectionism that interferes with task completion, excessive devotion to work and productivity to the exclusion of leisure activities, and preoccupation with details, rules, lists, order, organization, and schedules (American Psychiatric Association (APA) 2013). OCPD is closely related to depression, since its prevalence in patients with 12-month mood disorders is 24% (Grant et al. 2012). Moreover, OCPD accelerates relapse into new depressive episodes (Grilo et al. 2010).

Pregnancy and the postpartum period are characterized by many changes and adaptations, most of which are outside the woman’s control (e.g., bodily changes and physical complaints during pregnancy; the sleeping and feeding pattern of the baby). Within a vulnerability-stress model, all these perinatal biological and psychosocial changes could trigger depressive symptoms in already vulnerable women (Riecher-Rössler and Hofecker Fallahpour 2003). In the case of vulnerability, coping with perinatal changes is likely to be particularly challenging for women who experience a strong need for control and predictability. As such, expectant mothers with OCPD may be at increased risk of depressive symptoms in the postpartum period. Up until now, OCPD has not been linked to trajectories of postpartum depressive symptoms. However, postpartum depression (PPD) is a heterogeneous disorder (Nandi et al. 2009) and the course of depressive symptoms over time is extremely variable (Santos et al. 2017). Therefore, in the current study, we used growth mixture modeling with five repeated assessments of depressive symptoms, in order to identify trajectories of depressive symptoms during the first year postpartum. Subsequently, we aimed to study the relationship of OCPD trait symptoms to these trajectories. Due to the heterogeneity of postpartum depression (Nandi et al. 2009; Santos et al. 2017), our first hypothesis was that we would identify various trajectories of depressive symptoms. In addition, due to the known association between OCPD and depression (Grilo et al. 2010; Grant et al. 2012; Diedrich and Voderholzer 2015), our second hypothesis was that higher levels OCPD trait symptoms would, at some point during the postpartum period, be independently associated with those trajectories exhibiting elevated depressive symptoms.

## Methods

### Participants and procedure

**Fig. 1:**
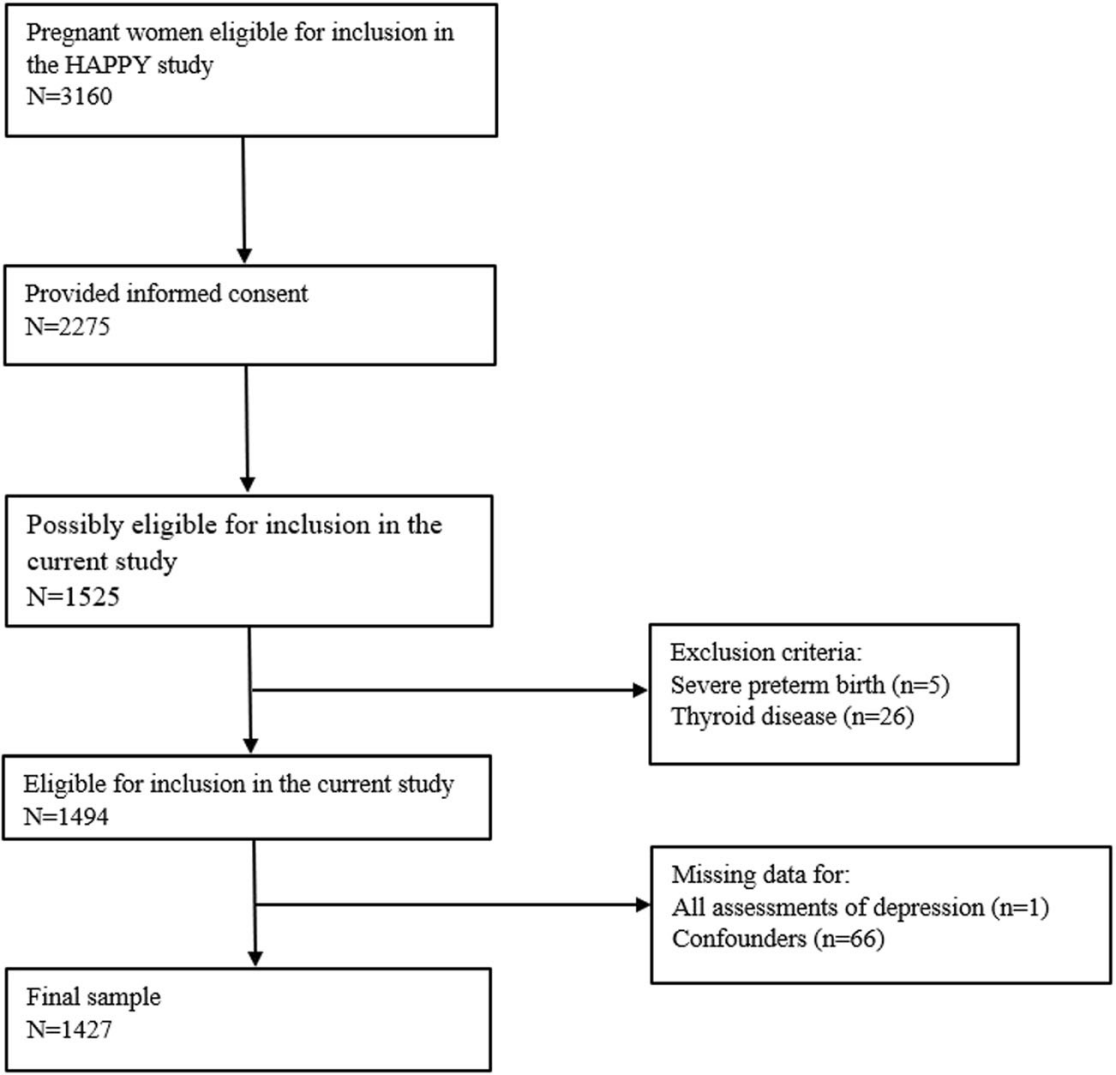
Flowchart of participant inclusion

The current study forms part of the Holistic Approach to Pregnancy and the first Postpartum Year (HAPPY) study, a large prospective cohort study, the design of which has already been described in detail elsewhere (Truijens et al. 2014). The Dutch obstetric care system is organized into primary care for low-risk pregnancies, represented by independent midwives, and secondary care for high-risk pregnancies, represented by hospital midwives and gynecologists. Management of 84% of all pregnant women starts in midwifery practices (The Netherlands Perinatal Registry (Perined) 2012). The remaining 16% of women are high risk, consisting of women with, for example, a chronic disease, psychiatric disorder, gemelli pregnancy. As we wished to address the low-risk population in the current study, women with high-risk features were excluded from participation and women were recruited by midwife practices. Women were recruited by 17 community midwife practices at the time of their first prenatal visit from January 2013 and September 2014. The main inclusion criterion was a singleton pregnancy. Due to the design of the current study, which relied heavily on Dutch-language questionnaires, only women with an advanced understanding of Dutch were eligible for inclusion. Exclusion criteria included known history of a severe psychiatric disorder with referral to a special outpatient policlinic for psychiatric pregnant patients (bipolar depression, personality disorder), and a previous diagnosis of a chronic condition (e.g., type 1 diabetes, thyroid disorder). In total, 3160 women were informed about the HAPPY study, 2275 (72%) of whom gave their informed consent. Since we chose to assess OCPD trait symptoms at a later stage of the HAPPY study, only 1525 participating women could be included in the current study. We excluded women with severe preterm birth (delivery at < 32 weeks of pregnancy) (*n* = 5) and a known thyroid disease at baseline (*n* = 26). Of the 1494 women remaining, in one case, data was missing on all assessments of depressive symptoms, and in 66 others, data was missing on confounders. All this ultimately resulted in a final sample of 1427 (96%) women. This final sample includes, among others, Dutch, Belgian, Iranian, Moroccan, Spanish, Hungarian, Russian, Portuguese, Chinese, Turkish, and Bangladeshi women (2.2% non-Dutch). The study was approved by the Psychology Ethics Committee of Tilburg University (protocol number EC-2012.25) and reviewed by the Medical Ethics Committee of the Máxima Medical Centre Veldhoven. All women provided written informed consent (Fig. 1).

#### Sample characteristics

At 12 weeks of pregnancy, we assessed several baseline sociodemographic, lifestyle, obstetric, and psychological characteristics (Table 1). With the level of education being high in 65% of the women, our sample was slightly more highly educated than the remainder of women in the HAPPY cohort, who were not included in the present study (60%; *p* = .02, *φ* = 0.05; very small effect size) (Cohen 1988), as well as compared to 49% of women aged between 25 and 35 years in the general Dutch population (Central Agency for Statistics Netherlands 2016). There was no difference in our sample compared to the remainder of the HAPPY cohort regarding age, parity, and the presence of a partner, but they were less likely to report their pregnancy as being unplanned or to have had a previous depressive episode (*p* = .02, *φ* = − 0.05; very small effect size) (Cohen 1988). Our study sample was comparable to other samples of (pregnant) women studied in the Netherlands (Bergink et al. 2011) and to data reported in the 2015 Netherlands Perinatal Registry with regard to obstetric parameters and mean age of the women (The Netherlands Perinatal Registry (Perined). Perinatale Zorg in Nederland 2015).

**Table 1:** Baseline characteristics of the women and results for study variables: depressive symptoms, OCPD trait symptoms, and confounders (*N* = 1427)

		*N* (%)	Mean (SD)	Range
Sociodemographic				
Age (years)			30.4 (3.7)	19–43
Educational level				
	Low/medium	504 (35.3)		
	High^a^	923 (64.7)		
Marital status				
	Partner	1407 (98.6)		
Paid job		1330 (93.2)		
Lifestyle				
BMI^c^			23.8 (3.9)	16.0–41.4
Alcohol use during pregnancy		27 (1.9)		
Smoking during pregnancy		60 (4.2)		
Obstetric				
Unplanned pregnancy		82 (5.7)		
Previous abortion/miscarriage		371 (26.0)		
Primiparous		705 (49.4)		
Psychological				
Previous depressive episode (s)		210 (14.7)		
OCPD trait symptoms at 32 weeks of pregnancy			8.7 (3.5)	0–21
Depressive symptoms^b^				
	32 wP		4.9 (4.2)	0–24
	6 wPP		4.9 (4.6)	0–25
	4 mPP		4.7 (4.5)	0–25
	8 mPP		4.6 (4.7)	0–26
	12 mPP		4.4 (4.3)	0–23

*SD* standard deviation, *BMI* body mass index, *OCPD* obsessive-compulsive personality disorder, *wP* weeks of pregnancy, *wPP* weeks postpartum, *mPP* months postpartum

^a^Bachelor or Master’s degree

^b^At 6 weeks and 4, 8 and 12 months postpartum 18.1, 20.3, 22.9, and 33.9% missing, respectively

### Measures

#### Depressive symptoms

Depressive symptoms during the past 7 days were assessed at 32 weeks of pregnancy and at 6 weeks and 4, 8, and 12 months postpartum using the 10-item Edinburgh (Postnatal) Depression Scale (E (P)DS) (Pop et al. 1992; Bergink et al. 2011). Item 10 focuses on suicidal ideation. Total scores range from 0 to 30, with the higher scores indicating higher levels of depressive symptoms. Postpartum, a cut-off value of ≥ 13 is often used as aid in the detection of a syndromal diagnosis of major depression (Gaynes et al. 2005) and can be considered a high score. In the present study, Cronbach’s α ranged from .84 to .88.

#### OCPD trait symptoms

OCPD trait symptoms were measured at 32 weeks of pregnancy using the seven-item Pregnancy OCPD Symptoms Checklist (van Broekhoven et al. 2017). The questionnaire addresses need for control, and the Diagnostic and Statistical Manual of Mental Disorders-Fifth Edition (DSM-5) OCPD criteria of perfectionism, preoccupation with details, and excessive devotion to work and productivity (APA 2013). Total scores range from 0 to 21, with higher scores indicating higher levels of OCPD trait symptoms. In the present study, Cronbach’s α was .77.

### Statistical analyses

#### Trajectories of postpartum depressive symptoms

Analyses were performed in Mplus version 7.4 (Muthén and Muthén 1998–2015). In order to determine the trajectories of postpartum depressive symptoms, we performed growth mixture modeling. We used the EPDS scores at 32 weeks’ pregnancy and at 6 weeks and 4, 8, and 12 months’ postpartum. The E (P) DS assessment at 32 weeks’ pregnancy was included in order to take into account the possible effect of high depressive symptoms when completing the Pregnancy OCPD Symptoms Checklist. Childbirth was chosen to be the Mplus (statistical) starting point for the development of postpartum depressive symptoms, and the spacing between measurement points matched the actual number of weeks in between time points (van de Schoot et al. 2017). Missing data on the EPDS were handled in full information maximum-likelihood (FIML) estimates ( Muthén and Muthén 1998–2015). Since the EPDS scores were positively skewed with a large number of scores being equal to zero, we used a censored normal distribution as well as the MLR option (i.e., maximum likelihood estimation with robust standard errors). Models with linear (slope) only and both linear and quadratic growth factors were estimated and compared. The starting point was a one-class model, after which we fitted models with increasing numbers of classes. In the current study, each class represented a trajectory of postpartum depressive symptoms. In order to determine the optimal number of classes, we took the following fit indices into account: Bayesian Information Criterion (BIC), Lo-Mendell-Rubin Likelihood Ratio Test (LMR-LRT), and Bootstrapped likelihood ratio test (BLRT) (Nylund et al. 2007; Jung and Wickrama 2008). Better-fitting models have lower BIC values (Collins and Lanza 2010), and significant LMR-LRT and BLRT values indicate that a model with an additional class improves model fit. As well as these fit indices, we also considered entropy, with entropy values closer to 1 indicating clearer delineation of classes (Collins and Lanza 2010). Finally, we took parsimony and interpretability of the models into account. Once the trajectory classes were determined, women were assigned to their most likely class by Mplus while taking classification uncertainty into account (Vermunt 2010).

#### Examining OCPD trait symptoms in relation to trajectories of postpartum depressive symptoms

Using the three-step model ML approach (Vermunt 2010; Asparouhov and Muthén 2014), we examined the relationship between OCPD trait symptoms and the established postpartum depressive symptom trajectories through multinomial regression. Multinomial regression was used to predict a nominal dependent variable with more than two categories (i.e., our three trajectories of postpartum depressive symptoms) from a set of independent variables, where one of the categories of the dependent variable acts as a reference category (Hosmer et al. 2013). In the present study, the reference category was the trajectory that included the highest percentage of women. In order to assess the independent association of OCPD trait symptoms with the trajectories, we adjusted for several possible confounders: age, educational level, unplanned pregnancy, self-reported previous depressive episode(s), and parity (Beck 2001; O’Hara and McCabe 2013; Santos et al. 2017). Depressive symptoms during pregnancy had already been taken into account in the modeling of trajectories of postpartum depressive symptoms. Both unadjusted and adjusted odds ratios (ORs) were determined for OCPD trait symptoms, with corresponding 95% confidence intervals (CIs).

## Results

### Descriptive statistics

Descriptive statistics are presented in Table 1. Approximately 15% of women self-reported previous depressive episode(s), and 6% indicated that their pregnancy was unplanned. The mean depressive symptom scores reflect the low-risk status of the participating women. The Pearson r correlations between OCPD trait symptoms reported during pregnancy and depressive symptoms assessed at the four postpartum measurements ranged from .28 to .32 (*p* < .001, medium effect size) (Cohen 1988).

### Trajectories of postpartum depressive symptoms

We refer to the supplementary text (Online Resource 1) for an extensive discussion of the technical details of model decisions. We fitted growth mixture models (GMMs) (Muthén and Shedden 1999) with free but equal growth factor variances for the intercept and the slope and quadratic growth factor variances fixed at zero. Results regarding the optimal number of latent classes and additional information are presented in Table 2.

**Table 2:** Model fit indices for deciding on the number of classes of postpartum depressive symptoms: growth mixture models with linear (slope) and quadratic effects

Number of classes	BIC	LMR-LRT *p*	BLRT *p*	Entropy	*n*(%) of women in each class
1	29,156.61	–	–	–	1427 (100)
2	28,965.78	.02	<.001	.86	1318 (92.3), 109 (7.7)
*3*	*28,904.68*	*.04*	*<.001*	*.89*	*1309 (91.7), 77 (5.4), 41 (2.9)*
4	28,885.76	.16	<.001	0.88	1268 (88.8), 72 (5.1), 46 (3.2), 41 (2.9)

For each model, all classes had free (equal) intercept growth factor variances and the slope and quadratic growth factor variances fixed to zero. For the 1-, 2-, and 3-class models, the number of random starts was 100 and the number of final stage iterations was 20. For the 4-class model, the number of random starts was increased to 500. Values in italics indicate the final model

*BIC* Bayesian Information Criterion, *LMR-LRT* Lo-Mendell-Rubin Likelihood Ratio Test, *BLRT* Bootstrapped likelihood ratio test

**Fig. 2:**
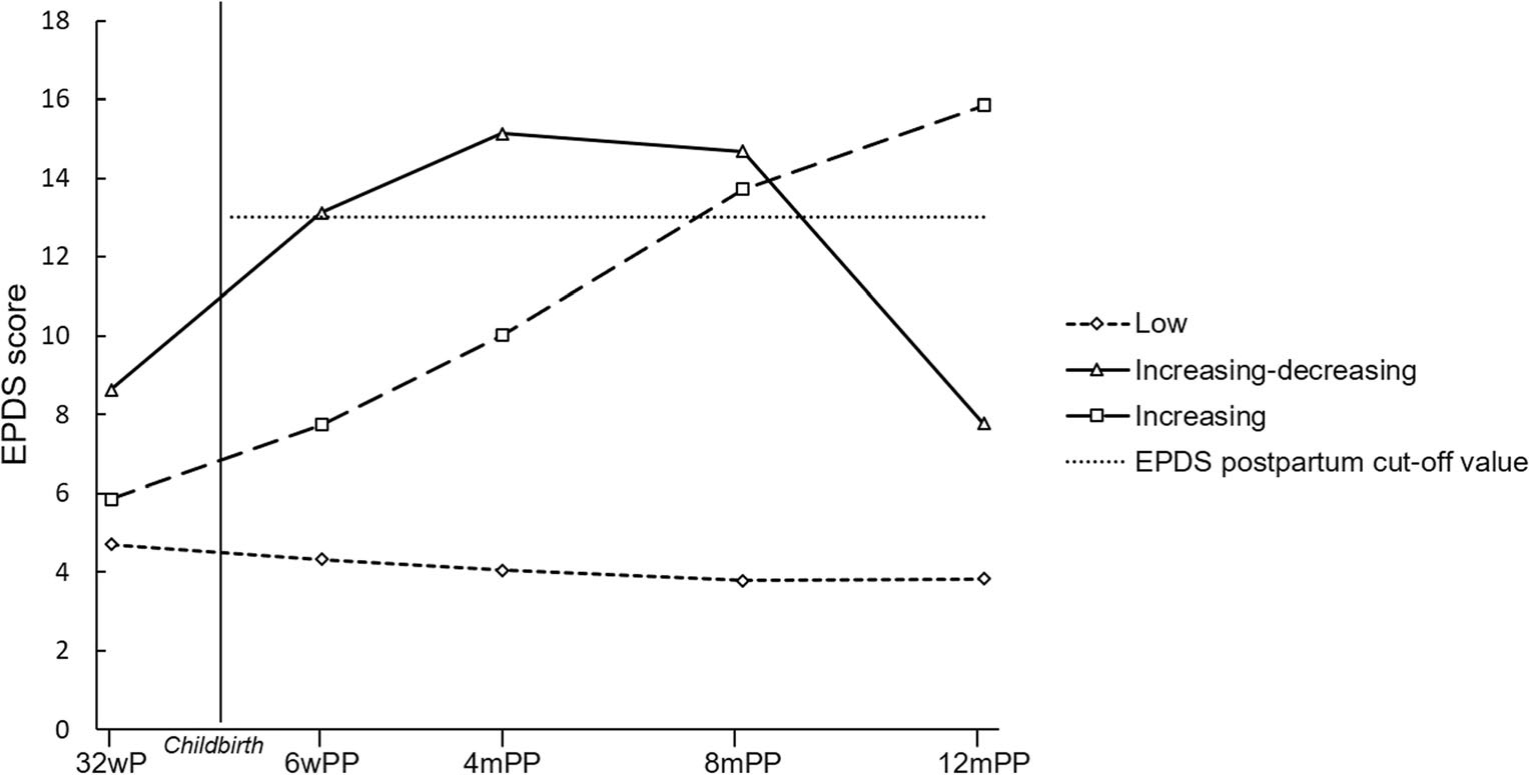
Three trajectories of postpartum depressive symptoms. wP weeks’ pregnancy; wPP weeks’ postpartum; mPP months’ postpartum; EPDS Edinburgh Postnatal Depression Scale

Based on the BIC, LMR-LRT, and BLRT statistics, the two-class GMM proved to be a significantly better fit than the one-class model and, in turn, the three-class model outperformed the two-class one. In the case of the four-class model, the BLRT remained significant but the LMR-LRT was rendered non-significant. Moreover, the BIC decrease was only small and substantially smaller than earlier decreases. In addition, the entropy value decreased slightly from the three- to the four-class model. For these reasons, and for the sake of parsimony and interpretability, the three-class model was chosen. This model had readily interpretable and clinically relevant trajectories, as well as adequate class sizes and entropy. The three trajectories are shown in Fig. 2.

The first class (*n* ≈ 92%) was labeled “*Low symptoms trajectory*” and consisted of women who reported low levels of depressive symptoms throughout the postpartum period (mean EPDS score range 3.8–4.7) and is regarded as the reference group. The second class (*n* ≈ 5%) was labeled “*Increasing-decreasing symptoms trajectory*” and consisted of women with high levels of postpartum depressive symptoms at 6 weeks and 4 and 8 months’ postpartum, which decreased to antenatal levels towards the end of the first postpartum year (inverted u-shape; mean EPDS score range 7.8–15.1). The third class (*n* ≈ 3%) was labeled “*Increasing symptoms trajectory*” and consisted of those women who reported an increasing level of postpartum depressive symptoms over time, with high scores at 8 and 12 months’ postpartum (mean EPDS score range 5.8–15.8). The parameter estimates are presented in Supplementary Table 1 (Online Resource 2).

#### OCPD trait symptoms in relation to trajectories of postpartum depressive symptoms

Unadjusted estimates for OCPD trait symptoms were OR 1.29, 95% CI 1.17–1.29 and OR 1.14, 95% CI 1.01–1.29 for the *Increasing-decreasing symptoms trajectory* and the *Increasing symptoms trajectory*, respectively, compared to the reference category. After adjusting for the confounders, OCPD trait symptoms were significantly and independently associated with the postpartum depressive symptom trajectory class membership. Higher levels of OCPD trait symptoms were associated with increased odds of being assigned to the *Increasing-decreasing symptoms trajectory* (OR 1.26, 95% CI 1.14–1.39, *p* < .001) and *Increasing symptoms trajectory* (OR 1.16, 95% CI 1.02–1.32, *p* = .02), compared to the odds of being part of the *Low symptoms trajectory* (reference category)*.* The association of OCPD trait symptoms with the *Increasing-decreasing symptoms trajectory* versus the *Increasing symptoms trajectory* was not significantly different (OR 1.08, 95% CI 0.92–1.27, *p* = 0.33). The results of the multinomial regression, including all confounders, are presented in Supplementary Table 2 (Online Resource 3). An odds ratio (OR) of 1.26 should be interpreted as follows: for every increase of 1 on the OCPD Symptoms Checklist (with scores ranging from 0 to 21), the odds of being part of the *Increasing-decreasing symptoms trajectory* compared to the reference category are 1.26 times more likely (i.e., increased odds of 26 percentage points). For example, this also implies that an increase of five points on the OCPD Symptoms Checklist corresponds to an increased odds of 1.26^5^ = 3.18 (or 218 percentage points) of being part of the *Increasing-decreasing symptoms trajectory*, compared to the reference group. Due to the substantial co-morbidity between OCPD and depression (Grant et al. 2012), we repeated the analysis without “previous depressive episode (s)” being included as a confounder, with similar results. The mean level of OCPD trait symptoms in the *Increasing-decreasing symptoms trajectory* (mean score 11.6, standard deviation (SD) 4.2) was significantly higher than in the reference group (mean score 8.5, SD 3.6, *p* < .001). There was no difference in the mean level of OCPD trait symptoms between the *Increasing symptoms trajectory* (mean score 9.9, SD 4.0) and the reference group (*p* = .05). A score of 11 or more corresponds approximately to the highest quartile of scores in the total sample.

When a score of ≥ 11 is considered a high OCPD trait symptoms score, 4.1% of women with high scores reported suicidal ideation at some point during the perinatal period, compared to 2.0% of those with lower scores (EPDS item 10 “The thought of harming myself has occurred to me”: Sometimes/Yes, quite often). The level of OCPD trait symptoms was higher in women with suicidal ideation (mean score 10.5, SD 4.7) compared to those who did not report suicidal ideation (mean score 8.6, SD 3.4), t(33.9) = −2.4, *p* = .02 (Cohen’s *d* = .47, medium effect size) (Cohen 1988).

## Discussion

### Three trajectories of depressive symptoms during the first postpartum year

We identified three trajectories of depressive symptoms during the first postpartum year, as follows: *Low symptoms*, *Increasing-decreasing symptoms*, and *Increasing symptoms*. Previously, in a similar sample of perinatal women followed during pregnancy and up to 12 months’ postpartum, Fredriksen et al. (2017) identified postpartum trajectories of minimum symptoms, moderate-persistent symptoms that increased as time passed after birth, and symptoms that were limited to either pregnancy or the early postpartum period. Our results showed that a large majority of women (≈ 92%) reported low levels of depressive symptoms throughout the postpartum period, which reflects the low-risk status of the women participating in this study. At the same time, a small but clinically relevant number of women (≈ 8%) experienced high levels of depressive symptoms at some point during the postpartum period. The present findings are in line with a recent review showing that perinatal trajectories with stable symptom levels over time (in the current study: stable with a low level of severity) are common, whereas less stable trajectories tend to be seen in a smaller number of women (Santos et al. 2017).

### OCPD trait symptoms increase the risk of postpartum depressive symptomatology

The current study is the first to report on the relationship between OCPD trait symptoms and trajectories of postpartum depressive symptoms until 12 months’ postpartum. These results were found after adjustment for several factors, such as previous depressive episode(s), thus underlining the added value of the Pregnancy OCPD Symptoms Checklist in the identification of women at risk for postpartum depressive symptoms. A previous study in a small sample suggested an association between personality disorders, including OCPD, and postpartum-onset depression (*N* = 302, OCPD *n* = 10), but this study did not take into account the heterogeneity of the disease course (Akman et al. 2007). In the present study, women with *increasing-decreasing* levels of depressive symptoms appeared to experience initial difficulties in adapting to the changes and challenges related to caring for a baby, but as time passed, these women adjusted and their depressive symptom levels decreased. In the women with *increasingly high* levels of depressive symptoms, challenges seemed to build up over time, and after an initial period with relatively mild levels of depressive symptoms, they showed high levels of depressive symptoms towards the end of the first postpartum year. OCPD trait symptoms are associated with both these developmental *patterns* of postpartum depressive symptoms (and not just with a single elevated depressive symptoms score), and the association with OCPD trait symptoms was particularly pronounced in those women with *increasing-decreasing* levels of depressive symptoms during the first postpartum year. Based on our findings, a Pregnancy OCPD Symptoms Checklist score of ≥ 11 (corresponding approximately to the highest quartile of scores in the total sample) could signal vulnerability, since it was associated with high levels of depressive symptoms during the first postpartum year. Future research should perhaps focus on identifying the adequate cutoff value for the Pregnancy OCPD Symptoms Checklist in relation to postpartum depressive symptoms.

### Strengths and limitations

The present study was the first to focus on OCPD trait symptoms in relation to trajectories of postpartum depressive symptoms. Some of its strengths are its large sample size and prospective design with five repeated assessments, which enabled us to use growth mixture modeling (Nagin 2005). This statistical method takes into account the heterogeneous development of depressive symptoms over time, which cannot adequately be captured using a *one-size-fits-all* approach (Santos et al. 2017). Growth mixture modeling is substantially different from conventional growth modeling techniques, which assume that individuals form a homogeneous group and that a single growth trajectory adequately reflects all group members (Jung and Wickrama 2008). Based on multiple assessments, growth mixture modeling is able to uncover various developmental patterns of depressive symptoms, simultaneously taking all subsequent assessments into consideration. This corresponds considerably better to perinatal clinical practice, where large individual differences in the development of depressive symptoms over time can be seen (Segre and Davis 2013). In addition, contrary to cross-sectional analyses, the developmental patterns of postpartum depressive symptoms are true longitudinal patterns, such that they include the same women at each time point. The women in our sample adequately reflect perinatal women in the Netherlands with regard to obstetric parameters and mean age ((Perined) 2015). Several study limitations should also be acknowledged. First, we assessed depressive symptoms with the self-report questionnaire EPDS (Cox et al. 1987) rather than carrying out a diagnostic psychiatric interview. Therefore, high-intensity depressive symptom scores should be interpreted with some caution. Secondly, the Pregnancy OCPD Symptoms Checklist does not cover all DSM-5 OCPD criteria (APA 2013). However, components of the defining features of OCPD, according to DSM-5 (i.e., orderliness, perfectionism, and control) (APA 2013), were covered by our instrument. Although other scales that encompass OCPD items do exist (Samuel and Widiger 2010), the Pregnancy OCPD Symptoms Checklist is the only instrument specifically developed for, and validated in, a sample of pregnant women (van Broekhoven et al. 2017). Moreover, we studied a low-risk population of relatively highly educated, predominantly Caucasian, all Dutch-speaking, women most of whom had a partner and, as a result, our findings may be limited in their generalizability (e.g., to different ethnic minorities). At the same time, our findings indicate that OCPD trait symptoms are a vulnerability factor in a subgroup of this population. It should be noted that women with a known history of a severe psychiatric disorder, having been referred to a special outpatient policlinic for psychiatric pregnant patients, were excluded from this study. Therefore, our trajectories of severe postpartum depressive symptoms were found in a healthy general population of pregnant women. Another limitation is that we did not take account of partner support in the present study, since meta-analyses have shown that poor partner support and social support have moderate to strong associations with postpartum depression (O’Hara and McCabe 2013). Nevertheless, we did include the following risk factors for postpartum depression: age, educational level, unplanned pregnancy, self-reported previous depressive episode(s), and parity. A final remark, related to the heterogeneity of depression, should be made. While the present study focused on depression as a primarily affective disorder, the wide variety of possible disturbances in cognition should be taken into account as well. Cognitive impairments during depression are manifold and affect both elementary and more complex cognitive processes (Gonda et al. 2015). Persistent cognitive dysfunction is also important clinically since it has been found to decrease coping capacities (Castaneda et al. 2008), which are of course important to women who have recently given birth.

### Clinical implications and future research

Ideally, the identification of vulnerability factors that increase the risk of postpartum depressive symptoms should be started during pregnancy (Boyce 2003). Beyond the classical risk factors for PPD, such as past psychopathology (O’Hara and McCabe 2013), personality traits should not be overlooked (Boyce et al. 2001) and should be part of a postpartum depression risk profile. In line with this, what the present study is striving to make clear is that OCPD trait symptoms assessed during pregnancy are independently associated with postpartum depressive symptoms. Future research needs to study the association between OCPD and postpartum depression more thoroughly by means of, for example, diagnostic interviews. This could be combined with a closer look at individual symptoms of depression, such as suicidal ideation, in relation to OCPD trait symptoms. This is important since suicidal ideation is associated with significant suicidality as evidenced from item 10 of the EPDS (“The thought of harming myself has occurred to me”) (Howard et al. 2011). Although perinatal women appear to be less likely to act upon suicidal ideas compared to non-pregnant populations (Lindahl et al. 2005), more information on suicidal ideation and behavior in perinatal depressive women with OCPD traits is required. In the present study, the presence of postpartum suicidal ideation was more than doubled in women with high rather than lower levels of OCPD trait symptoms, which underlines the diagnostic value of the Pregnancy OCPD Symptoms Checklist for detecting women at risk of postpartum depressive symptoms. Health professionals should be aware of the possible presence of OCPD trait symptoms in pregnant women, since these symptoms are associated with an increased likelihood of high levels of depressive symptoms during the first year postpartum. Pregnancy would be the ideal time to start identifying OCPD trait symptoms since women are regularly in contact with health professionals at this time. Assessing OCPD trait symptoms during pregnancy with the Pregnancy OCPD Symptoms Checklist could aid in the identification of the subgroup of women vulnerable for high levels of postpartum depressive symptoms. The Pregnancy OCPD Symptoms Checklist is easy to administer and only needs to be assessed once during pregnancy, since we have previously been able to show that OCPD trait symptoms measured with the checklist are stable over time (van Broekhoven et al. 2017). As a next step, the development or course of depressive symptoms in these women should be carefully monitored. If necessary, they could then be offered interventions targeted at alleviating depressive symptoms and OCPD trait symptoms. Research has shown that cognitive therapy is effective in reducing symptoms of both OCPD and depression (Diedrich and Voderholzer 2015), but insight into the effectiveness of psychological therapies in perinatal women is needed. Attention should also be paid to a woman’s unique constellation of OCPD trait symptoms, such as aiming to be a perfect mother for her baby in all possible ways, or striving to be in control of everything, at the expense of her own well-being.

## Electronic supplementary material

Online Resource 1 (PDF 133 kb)

Online Resource 2 (PDF 73 kb)

Online Resource 3 (PDF 72 kb)

## References

[CR1] Akman C, Uguz F, Kaya N (2007). Postpartum-onset major depression is associated with personality disorders. Compr Psychiatry.

[CR2] American Psychiatric Association (2013). Diagnostic and statistical manual of mental disorders.

[CR3] Asparouhov T, Muthén B (2014). Auxiliary variables in mixture modeling: three-step approaches using Mplus. Struct Equ Model.

[CR4] Beck CT (2001). Predictors of postpartum depression: an update. Nurs Res.

[CR5] Bergink V, Kooistra L, Lambregtse-van den Berg MP, Wijnen H, Bunevicius R, van Baar A, Pop V (2011). Validation of the Edinburgh depression scale during pregnancy. J Psychosom Res.

[CR6] Boyce PM (2003). Risk factors for postnatal depression: a review and risk factors in Australian populations. Arch Womens Ment Health.

[CR7] Boyce P, Hickey A, Gilchrist J, Talley NJ (2001). The development of a brief personality scale to measure vulnerability to postnatal depression. Arch Womens Ment Health.

[CR8] Castaneda AE, Suvisaari J, Marttunen M, Perala J, Saarni SI, Aalto-Setala T, Aro H, Koskinen S, Lonnqvist J, Tuulio-Henriksson A (2008). Cognitive functioning in a population-based sample of young adults with a history of nonpsychotic unipolar depressive disorders without psychiatric comorbidity. J Affect Disord.

[CR9] Central Agency for Statistics Netherlands (2016) CBS StatLine – Bevolking*; hoogstbehaald onderwijsniveau en onderwijsrichting*. http://statline.cbs.nl/Statweb/publication/?DM=SLNL&PA=82816NED&D1=0&D2=l&D3=3&D4=0&D5=0-1,7,11&D6=0&D7=69&HDR=G6,G3,G2,G4,G1&STB=T,G5&VW=T Accessed 19 December 2017

[CR10] Cohen JW (1988). Statistical power analysis for the behavioural sciences.

[CR11] Collins LM, Lanza ST (2010). Latent class and latent transition analysis: with applications in the social, behavioral, and health sciences.

[CR12] Cox JL, Holden JM, Sagovsky R (1987). Detection of postnatal depression. Development of the 10-item Edinburgh postnatal depression scale. Br J Psychiatry.

[CR13] Diedrich A, Voderholzer U (2015). Obsessive–compulsive personality disorder: a current review. Curr Psychiatry Rep.

[CR14] Fredriksen E, von Soest T, Smith L, Moe V (2017). Patterns of pregnancy and postpartum depressive symptoms: latent class trajectories and predictors. J Abnorm Psychol.

[CR15] Gaynes BN, Gavin N, Meltzer-Brody S, Lohr KN, Swinson T, Gartlehner G, Brody S, Miller WC (2005) Perinatal depression: prevalence, screening accuracy, and screening outcomes. Summary, Evidence Report/Technology Assessment No 119. Agency for Healthcare Research and Quality, Rockville, MD 10.1037/e439372005-001 PMC4780910 15760246

[CR16] Gonda X, Pompili M, Serafini G, Carvalho AF, Rihmer Z, Dome P (2015). The role of cognitive dysfunction in the symptoms and remission from depression. Ann General Psychiatry.

[CR17] Grant JE, Mooney ME, Kushner MG (2012). Prevalence, correlates, and comorbidity of DSM-IV obsessive-compulsive personality disorder: results from the National Epidemiologic Survey on alcohol and related conditions. J Psychiatr Res.

[CR18] Grilo CM, Stout RL, Markowitz JC, Sanislow CA, Ansell EB, Skodol AE, Bender DS, Pinto A, Shea MT, Yen S, Gunderson JG, Morey LC, Hopwood C, McGlashan TH (2010). Personality disorders predict relapse after remission from an episode of major depressive disorder: a 6-year prospective study. J Clin Pyschiatry.

[CR19] Hosmer DW, Lemeshow S, Sturdivant RX (2013). Applied logistic regression.

[CR20] Howard LM, Flach C, Mehay A, Sharp D, Tylee A (2011). The prevalence of suicidal ideation identified by the Edinburgh postnatal depression scale in postpartum women in primary care: findings from the RESPOND trial. BMC Pregnancy Childbirth.

[CR21] Jung T, Wickrama KAS (2008). An introduction to latent class growth analysis and growth mixture modeling. Soc Personal Psychol Compass.

[CR22] Lindahl V, Pearson J, Colpe L (2005). Prevalence of suicidality during pregnancy and the postpartum. Arch Womens Ment Health.

[CR23] Muthén LK, Muthén BO (1998-2015) Mplus User’s Guide, Seventh edn. Muthén & Muthén, Los Angeles, CA

[CR24] Muthén B, Shedden K (1999). Finite mixture modeling with mixture outcomes using the EM algorithm. Biometrics.

[CR25] Nagin DS (2005). Group-based modeling of development.

[CR26] Nandi A, Beard JR, Galea S (2009). Epidemiologic heterogeneity of common mood and anxiety disorders over the lifecourse in the general population: a systematic review. BMC Psychiatry.

[CR27] Nylund KL, Asparouhov T, Muthén B (2007). Deciding on the number of classes in latent class analysis and growth mixture modeling: a Monte Carlo simulation study. Struct Equ Model.

[CR28] O’Hara MW, McCabe JE (2013). Postpartum depression: current status and future directions. Annu Rev Clin Psychol.

[CR29] Pop VJ, Komproe IH, van Son MJ (1992). Characteristics of the Edinburgh post natal depression scale in the Netherlands. J Affect Disord.

[CR30] Riecher-Rössler A, Hofecker Fallahpour M (2003). Postpartum depression: do we still need this diagnostic term?. Acta Psychiatr Scand.

[CR31] Samuel DB, Widiger TA (2010). A comparison of obsessive-compulsive personality disorder scales. J Pers Assess.

[CR32] Santos H, Tan X, Salomon R (2017). Heterogeneity in perinatal depression: how far have we come? A systematic review. Arch Womens Ment Health.

[CR33] Segre LS, Davis WN (2013) Postpartum depression and perinatal mood disorders in the DSM. Postpartum Support International. http://www.postpartum.net/wp-content/uploads/2014/11/DSM-5-Summary-PSI.pdf. Accessed 27 November 2017

[CR34] The Netherlands Perinatal Registry [Stichting Perinatale Registratie Nederland]: Perinatal Care in the Netherlands 2012 [Perinatale Zorg in Nederland 2012]. Utrecht: The Netherlands Perinatal Registry; 2013

[CR35] The Netherlands Perinatal Registry (Perined). Perinatale Zorg in Nederland 2015. Perined, Utrecht

[CR36] Truijens SE, Meems M, Kuppens SM, Broeren MA, Nabbe KC, Wijnen HA, Oei G, van Son MJM, Pop VJM (2014). The HAPPY study (holistic approach to pregnancy and the first postpartum year): design of a large prospective cohort study. BMC Pregnancy Childbirth.

[CR37] van Broekhoven KEM, Karreman A, Hartman EE, Pop VJM (2017). Stability of the pregnancy obsessive-compulsive personality disorder symptoms checklist. Arch Womens Ment Health (online first).

[CR38] van de Schoot R, Sijbrandij M, Winter SD, Depaoli S, Vermunt JK (2017). The GRoLTS-checklist: guidelines for reporting on latent trajectory studies. Struct Equ Model.

[CR39] Vermunt JK (2010). Latent class modeling with covariates: two improved three-step approaches. Polit Anal.

