# Supplementary material for: Obsessive-compulsive personality disorder symptoms as a risk factor for postpartum depressive symptoms
Source: Arch Womens Ment Health. 2018 Aug 31;22(4):475–83. doi: 10.1007/s00737-018-0908-0 (PMC6647494; doi:10.1007/s00737-018-0908-0)
Supplement: Supplementary file 1 — (PDF 133 kb) [file 737_2018_908_MOESM1_ESM.pdf]

ONLINE RESOURCE 1: SUPPLEMENTARY TEXT

Longitudinal mixture modeling: technical details regarding the method and statistical analyses

The measurement point of 32 weeks of pregnancy was given a negative factor loading which located the start of the development of postpartum depressive symptoms at childbirth (the coding of time for the subsequent measurement point was as follows: -8, 6, 16, 32, 48[1]). The starting point of our analysis was the simplest longitudinal mixture model: latent class growth analysis (LCGA; Nagin, 2005). Using LCGA, homogeneity within each class is assumed, which means that growth factor variance and covariance estimates are fixed to zero within the classes (Kreuter and Muthén 2008). If the trajectory classes show great variability regarding the individual growth curves, and the trajectories require estimated growth variances, LCGA is not deemed suitable (van de Schoot et al. 2017) and growth mixture modeling should be considered. The growth mixture model (GMM) is an extension of LCGA, in that it also identifies distinct trajectories, and additionally allows individuals to vary around their trajectory-specific mean (Jung and Wickrama 2008). As such, the GMM allows within-class variation of individuals (Muthén and Muthén 2000) which can provide a more realistic representation of complex data (Muthén 2006).

Models with linear (slope) only and both linear and quadratic growth factors, were estimated and compared. Inspection of the visual and numeric data showed significant quadratic growth, and Bayesian Information Criterion (BIC) values were more optimal in the models that included quadratic growth. For these reasons, models with both linear and quadratic growth were chosen in favor of the models with linear growth only. Visual inspection of the observed individual growth curves of these models indicated substantial deviation from the mean class trajectories. Therefore, we continued with GMMs with free (but equal across classes) intercept and slope variances (Muthén 2006), and the quadratic variance fixed to zero in each class. When we compared these models to GMMs with free (but equal across classes) intercept variance only, and with the slope and quadratic variance fixed to zero in each class, both these approaches resulted in similar BICs. For this reason, we continued with the more parsimonious models with free (but equal across classes) variances for the intercept growth factor only. These models had substantially better BIC values and superior entropy values compared to the simpler LCGAs. Models with *class specific* free intercept variances were investigated as a last step, but did not lead to better model fit compared to the models with free but *equal* variances (in fact, BIC was slightly less optimal for the models with class-specific free variances). We therefore decided on the GMMs with free but equal intercept growth factor variance across classes, and with the slope

---

[1]As the average duration of pregnancy is 40 weeks, we assigned the measurement point of 32 weeks of pregnancy a negative loading of -8, since it is located approximately eight weeks prior to childbirth. In this line, the measurement point of six weeks postpartum is located six weeks after childbirth and, as such, was given a loading of +6. We used these statistical loadings in Mplus to put the starting point (*i.e.*, 0) of postpartum depressive symptoms at childbirth (*i.e.*, at 40 weeks of pregnancy).

and quadratic variances fixed to zero. The main results are reported in the body of this paper. Apart from the fit indices, entropy, and parsimony and interpretability of the models, we also took the average posterior probabilities (APPs) of their classes into account. Posterior probability is the probability of membership in a specific class, given a certain response pattern (Collins and Lanza 2010), and should be at least .70 but rather .80 or higher (Nagin 2005). APPs of the final model were high (.80-.97). Using convergence checks, we were able to replicate the model estimates, which proved that the results of our final model were not a local solution (Jung and Wickrama 2008). This process involves increasing the number of starting values and final stage iterations in Mplus, in order to ensure that the solution converges on the global maximum solution (*i.e.*, the parameter estimates associated with the best loglikelihood) (Jung and Wickrama 2008).

References

Collins LM, Lanza ST (2010) Latent class and latent transition analysis: With applications in the social, behavioral, and health sciences. John Wiley & Sons, Hoboken

Jung T, Wickrama KAS (2008) An introduction to latent class growth analysis and growth mixture modeling. Soc Pers Psychol Compass 2:302–317. http://doi.org/10.1111/j.1751-9004.2007.00054.x

Muthén B, Muthén LK (2000) Integrating person-centered and variable-centered analyses: Growth mixture modeling with latent trajectory classes. Alcohol Clin Exp Res 24:882-891. http://doi.org/10.1111/j.1530-0277.2000.tb02070.x

Muthén B (2006) The potential of growth mixture modelling. Commentary. Infant Child Dev 15:623-625. http://doi.org/10.1002/icd.482

Nagin DS (2005) Group-based Modeling of Development. Harvard University Press, Cambridge, MA

Kreuter F, Muthén B (2008) Analyzing criminal trajectory profiles: Bridging multilevel and group-based approaches using growth mixture modeling. J Quant Criminol 24:1-31. http://doi.org/10.1007/s10940-007-9036-0

van de Schoot R, Sijbrandij M, Winter SD, Depaoli S, Vermunt JK (2017) The GRoLTS-Checklist: Guidelines for Reporting on Latent Trajectory Studies. Struct Equ Modeling 24:451-467. https://doi.org/10.1080/10705511.2016.1247646

Obsessive-compulsive personality disorder symptoms as a risk factor for postpartum depressive symptoms

Archives of Women's Mental Health

Kiki E.M. van Broekhoven, Annemiek Karreman, Esther E. Hartman, Paul Lodder, Joyce J. Endendijk, Veerle Bergink, Victor J.M. Pop[1]. [1]Corresponding author. Email address: v.j.m.pop@uvt.nl. Department of Medical and Clinical Psychology, Tilburg University.
