# Supplementary material for: Obsessive-compulsive personality disorder symptoms as a risk factor for postpartum depressive symptoms
Source: Arch Womens Ment Health. 2018 Aug 31;22(4):475–83. doi: 10.1007/s00737-018-0908-0 (PMC6647494; doi:10.1007/s00737-018-0908-0)
Supplement: Supplementary file 2 — (PDF 73 kb) [file 737_2018_908_MOESM2_ESM.pdf]

**ONLINE RESOURCE 2: SUPPLEMENTARY TABLE 1.**

Supplementary Table 1. Parameter estimates and average posterior probabilities for the three trajectories of postpartum depressive symptoms.

| Trajectory of postpartum depressive symptoms | N (%) | APP | Parameters | | B (SE) |
|---|---|---|---|---|---|
| 1. Low symptoms trajectory | 1309 (91.7) | .97 | | | |
| | | | *Mean* | | |
| | | | | Intercept | 4.08 (0.17)*** |
| | | | | Linear | -0.03 (0.006)*** |
| | | | | Quadratic | 0.00 (0.00)** |
| | | | *Variance* | | |
| | | | | Intercept | 11.00 (1.09)*** |
| 2. Increasing-decreasing symptoms trajectory | 77 (5.4) | .80 | | | |
| | | | *Mean* | | |
| | | | | Intercept | 11.93 (0.73)*** |
| | | | | Linear | 0.38 (0.08)*** |
| | | | | Quadratic | -0.01 (0.002)*** |
| | | | *Variance* | | |
| | | | | Intercept | 11.00 (1.09)*** |
| 3. Increasing symptoms trajectory | 41 (2.9) | .83 | | | |
| | | | *Mean* | | |
| | | | | Intercept | 7.07 (0.99)*** |
| | | | | Linear | 0.20 (0.09)* |
| | | | | Quadratic | 0.00 (0.002) |
| | | | *Variance* | | |
| | | | | Intercept | 11.00 (1.09)*** |

APP, average posterior probability; SE, standard error. For the intercept growth factor, the variance was estimated but held equal across classes. For the linear (slope) and quadratic growth factors, the variance was fixed at zero in each class. *p<.05, **p<.01, ***p<.001.

**Obsessive-compulsive personality disorder symptoms as a risk factor for postpartum depressive symptoms**

*Archives of Women's Mental Health*

Kiki E.M. van Broekhoven, Annemiek Karreman, Esther E. Hartman, Paul Lodder, Joyce J. Endendijk, Veerle Bergink, Victor J.M. Pop[1]. [1]Corresponding author. Email address: v.j.m.pop@uvt.nl. Department of Medical and Clinical Psychology, Tilburg University.
