# Supplementary material for: Obsessive-compulsive personality disorder symptoms as a risk factor for postpartum depressive symptoms
Source: Arch Womens Ment Health. 2018 Aug 31;22(4):475–83. doi: 10.1007/s00737-018-0908-0 (PMC6647494; doi:10.1007/s00737-018-0908-0)
Supplement: Supplementary file 3 — (PDF 72 kb) [file 737_2018_908_MOESM3_ESM.pdf]

ONLINE RESOURCE 3: SUPPLEMENTARY TABLE 2.

Supplementary Table 2. Complete multinomial regression for trajectories of postpartum depressive symptoms.

	B (SE)	OR (95% CI)
Increasing-decreasing vs. Low symptoms		
OCPD trait symptoms	0.23 (0.05)	1.26 (1.14-1.39)***
Age	-0.05 (0.05)	0.96 (0.87-1.05)
Low-medium educational level	0.25 (0.35)	1.29 (0.65-2.55)
Unplanned pregnancy	-0.51 (0.96)	0.60 (0.09-3.93)
Previous depressive episode(s)	1.10 (0.39)	3.00 (1.39-6.47)**
Primiparity	0.14 (0.36)	1.15 (0.57-2.32)
Increasing vs. Low symptoms		
OCPD trait symptoms	0.15 (0.07)	1.16 (1.02-1.32)*
Age	0.14 (0.08)	1.15 (0.99-1.34)
Low-medium educational level	0.65 (0.47)	1.92 (0.76-4.86)
Unplanned pregnancy[a]	-	-
Previous depressive episode(s)	0.65 (0.54)	1.92 (0.66-5.55)
Primiparity	-0.06 (0.49)	.94 (0.36-2.43)

SE, standard error; OR, odds ratio; CI, confidence interval. OCPD trait symptoms and age are expressed on a continuous scale; all other variables are dichotomous. *p<.05, **p<.01, ***p<.001. [a]Effect could not be estimated as none of the women belonging to the *Increasing symptoms trajectory* reported their pregnancy as being unplanned.

Obsessive-compulsive personality disorder symptoms as a risk factor for postpartum depressive symptoms

Archives of Women's Mental Health

Kiki E.M. van Broekhoven, Annemiek Karreman, Esther E. Hartman, Paul Lodder, Joyce J. Endendijk, Veerle Bergink, Victor J.M. Pop[1]. [1]Corresponding author. Email address: v.j.m.pop@uvt.nl. Department of Medical and Clinical Psychology, Tilburg University.
